# Langfristige Verlaufskontrolle nach einem Coxiellose-Ausbruch in einem Ziegenbestand

**DOI:** 10.1055/a-2762-6316

**Published:** 2026-04-24

**Authors:** Jens Böttcher, Britta Janowetz, Michaela Alex, Christina Ambros, Ursula Domes

**Affiliations:** 1 Tiergesundheitsdienst Bayern e.V.

**Keywords:** Q-Fieber, Ziegen, Impfung, Überwachung, Proben, Umgebung, Q fever, goats, vaccination, surveillance, samples, environment

## Abstract

Im Januar 2020 wurde *Coxiella (C.) burnetii* in einem kleinen Ziegenbestand nachgewiesen, nachdem ein Abort, ein lebensschwaches und ein missgebildetes Kitz beobachtet worden waren.

Alle Tiere ab einem Alter von 3 Monaten wurden bis Mai 2020 mit Coxevac grundimmunisiert (2x im Abstand von 3 Wochen) und im Herbst 2020 und im Mai 2021 revakziniert. Die Jungtiere wurden mit Ausnahme von 2023 jährlich grundimmunisiert. Die Überwachung erfolgte mit der qPCR-Untersuchung von Milchproben, Vaginal- und Präputial- (Genitaltupfer, GT), Nasen- (NT) und Umgebungstupfern (UT). Blut- und Milchproben wurden auf Phasen-(PhI, PhII)-spezifische Antikörper untersucht, um die Impfung zu kontrollieren. Daten von einer geimpften, seronegativen Herde wurden für Vergleichszwecke herangezogen.

Die Erregerlast in allen Probentypen sank bis Juni 2020 kontinuierlich ab. Schwach positive Proben wurden bis November (Milch), Dezember (GT) nachgewiesen. Zwei NT waren im Mai und Dezember 2021 schwach positiv (5 C.b./NT). Die Nachweisraten in UT waren bis April 2020 hoch (24/24), sie verhielten sich über den weiteren Verlauf des Jahres konstant (31/40) und fielen erst in den Folgejahren sehr langsam ab: 12/24 (2021), 5/16 (2022), 2/8 (2023), 1/8 (2024) und 0/8 (2025). Es wurden keine persistent-infizierten Ziegen ermittelt.

Unerwartet führte die Impfung der Altziegen zu einem signifikanten Anstieg der PhI-Titer, die PhII-Titer veränderten sich jedoch nicht. Im Gegensatz hierzu führte die Impfung von seronegativen Jungziegen in der Fall- und Altziegen in der Kontrollherde zu einem signifikanten Anstieg der PhII-Titer, während PhI-Antikörper kaum beobachtet wurden.

Die Impfung führte zu keiner kurzfristigen Kontrolle der Erregerausscheidung, daher ist die prophylaktische Impfung sehr wichtig. Umgebungsproben erfordern eine quantitative Bewertung, sie sind langfristig für die Überwachung freier Bestände wertvoll. Positive Ergebnisse der Umgebungsproben sollten mit NT oder GT überprüft werden. Genital- und NT sind gleichwertig, NT sind aber einfacher zu entnehmen.

## Abstract (en)

In January 2020 *Coxiella (C.) burnetii* was detected in a small goat flock after the observation of a weak born kid, an abortion as well as a malformed kid.

All animals older than 3 months were subsequently primarily vaccinated with Coxevac (2x three weeks apart) until May 2020 and were revaccinated once in October/November 2020 and in May 2021. Young animals were primarily vaccinated annually except for 2023. Surveillance was performed by qPCR-testing of milk samples, vaginal and preputial (genital swabs, GT), nasal (NT) and environmental swabs (UT). Vaccination was controlled by blood and milk samples which were analyzed for phase-(PhI, PhII)-specific antibodies. Data were compared with those that had been collected in a seronegative goat herd in the course of vaccination.

The load of *C. burnetii* steadily decreased in all types of samples until June 2020. Weak positive samples were detected until November (milk) and December 2020 (GT). Two NT tested weak-positive (5 C.b./NT) in May and December 2021. No persistent infection was observed. The detection rate in UT was high until April 2020 (24/24), remained stable during the rest of the year (31/40) and slowly decreased during the following years: 12/24 (2021), 5/16 (2022), 2/8 (2023), 1/8 (2024), and 0/8 (2025).

Unexpectedly, vaccination only significantly increased the PhI-titers while PhII-titers remained stable. In contrast, in seronegative kids in the case- and adults in the control-herd vaccination induced a significant increase of PhII- titers while almost no PhI- titers were observed.

No short-term effect of vaccination was observed, therefore prophylactic vaccination is very important. A quantitative interpretation is required for environmental samples, these samples are valuable to verify the lack of infection. Positive results may be confirmed by NT or GT. NT are equivalent to GT, and they are collected more easily.

## Einleitung

Erkrankungen aufgrund einer Infektion mit *Coxiella (C.) burnetii* werden bei Menschen als Q-Fieber (‚query fever‘) und bei Tieren als Coxiellose bezeichnet, weil das Leitsymptom Fieber bei Tieren nicht beobachtet wird. *Coxiella burnetii* ist ein Zoonoseerreger. Kleine Wiederkäuer sind eine wesentliche Infektionsquelle für Menschen. Wiederholt wurden sogenannte Kleinraumepidemien bei Menschen auf kleine Wiederkäuer zurückgeführt 1 . Der bislang größte Q-Fieber-Ausbruch trat von 2007–2011 in den Niederlanden auf. Es wurden gut 4000 klinische Fälle bei Menschen registriert, das Ausmaß an Infektionen wurde auf mehr als 40 000 geschätzt 2 . Infektionen bei Ziegen gehen im Vergleich zu Schafen und Rindern mit einem erhöhten Abortrisiko einher 3 , 4 . Coxiellen haben einen ausgesprochenen Tropismus für den tragenden Uterus. Sie vermehren sich in Trophoblasten und werden in hoher Konzentration mit dem Fruchtwasser ausgeschieden 5 , 6 . Erregernachweise kommen durchaus bei 2 aufeinanderfolgenden Geburten vor, d. h. Coxiellen haben Strategien entwickelt, länger im Wirt zu persistieren 4 . So werden Coxiellen bei Milchkühen über Jahre (teilweise lebenslang!) in der Milch nachgewiesen, diese Persistenz geht mit hohen Antikörpertitern gegen die O-Antigene des Lipopolysaccharids (LPS, Phase I-Antigen) einher 7 8 9 . Coxiellen werden auch geburtsunabhängig und in geringer Konzentration im Kot, Urin und im Vaginalschleim nachgewiesen, allerdings wird vermutet, dass das Risiko einer Kontamination dieser Materialien aus dem Umfeld relativ groß ist 10 . In Hinblick auf die Abortdiagnose sollten Vaginaltupfer daher vorzugsweise zeitnah zur Geburt entnommen werden, ein derartiger Tupfer spiegelt dann die Erregerlast im Fruchtwasser wider 11 . Die hohe Erregerausscheidung während der Lammung führt zu einer massiven Kontamination der Stallumgebung. Insofern sind Umgebungsproben geeignet, das hieraus resultierende Risiko und die Verbreitung des Erregers abzuschätzen 12 , 13 . Infektiosität im Sinne von vermehrungsfähigen Coxiellen konnte in einer geimpften Ziegenherde noch bis zur 3. Lammsaison nach dem Ausbruch in Umgebungsproben nachgewiesen werden 14 . In einer geimpften Schafherde deuteten Serokonversionen in einer Sentinelgruppe bis zur 4. Lammsaison nach dem Ausbruch auf eine Restinfektiosität in der Umgebung hin 12 , 15 .

Die Impfung mit einem inaktivierten Phase I-Impfstoff (Coxevac, Ceva) führt nicht zu einer sterilen Immunität; aber sie senkt das Risiko der Erregerausscheidung und reduziert die ausgeschiedene Erregerlast 16 . Ein Monitoring der Erregerausscheidung nach der Impfung ist daher sicherlich sinnvoll. Die Infektion erfolgt i.d.R. über die Atemwege 17 . Unter der Annahme, dass Umgebungsstaub eingeatmet wird, sollten die Nachweise von Coxiellen auf der Nasenschleimhaut die Umfeldbelastung widerspiegeln. Diesen Ansatz publizierten Bauer et al. (2020) erstmalig für Ziegen 18 . Bereits 2013 begannen Untersuchungen zur Eignung von Nasentupfern bei Ziegen und Schafen 15 , 19 , 20 . In diesen Studien korrelierten die positiven Nachweise in Nasentupfern auf Herdenebene sehr gut mit jenen in Vaginaltupfern, die während der Lammung entnommen wurden.

Die serologische Diagnostik des Q-Fiebers basiert in der Humanmedizin auf dem Nachweis Phasen-spezifischer Antikörper 17 . Bei virulenten Erregern liegt ein komplettes LPS vor (PhI), nach wiederholter kultureller Passage gehen die O-Antigene des LPS sukzessive verloren, bis schließlich ein Erreger in PhII vorliegt 21 , 22 . Die O-Antigene des LPS sind das PhI- und die Gesamtheit der Antigene (u. a. Proteine) des PhII-Bakteriums sind das PhII-Antigen 23 . Nach einer Infektion des Menschen werden zunächst PhII-Antikörper und mit zeitlicher Verzögerung können auch PhI-Antikörper auftreten. Hohe PhI-Titer gelten als Hinweis auf eine chronische Infektion 24 . Nach einer experimentellen Infektion bildeten tragende Ziegen zunächst PhII- und mit deutlicher zeitlicher Verzögerung auch PhI-Antikörper 25 , eine Dominanz der PhII-Antikörper wurde auch im Rahmen akuter Ausbrüche gesehen 26 , 27 . Hohe PhI-Titer sind bei Milchkühen ein Hinweis auf eine persistente Infektion 7 , 9 .

Im vorliegenden Fall wird ein Coxiellose-Ausbruch bei Ziegen im Verlauf über 6 Jahre beschrieben.

## Material und Methoden

### Ziegenherden

Die Untersuchungen wurden in zwei Ziegenherden, einer Fall- und einer Kontrollherde, durchgeführt.

In der Fallherde mit ca. 24 weißen deutschen Edelziegen, 3 Zuchtböcken und der Nachzucht wurde im Januar 2020 eine Coxiellose diagnostiziert: Nach der Geburt eines lebensschwachen Kitzes und eines Abortes am 1. und 3. Januar 2020 wurde am 7. Januar 2020 ein missgebildetes Kitz geboren, das euthanasiert wurde. Das missgebildete Kitz und Vaginaltupfer der Mutter sowie der beiden anderen betroffenen Ziegen wurden zur Untersuchung eingeschickt. Bei dem missgebildeten Kitz lag eine rechtsseitige Anophthalmie und ein Hydrocephalus internus vor. In der Lunge des Kitzes wurden 10 ^1,3^ C.b./mg nachgewiesen. Die Vaginaltupfer der Muttertiere (Ziege 1–3) waren in der PCR positiv (10 ^7,9^ , 10 ^5,9^ , 10 ^5,8^ C.b./Tpf.). Das Kitz wurde mit negativem Ergebnis auf BTV, SBV, Chlamydia spp. und *Neospora caninum* untersucht. Die Geburtsjahrgänge der Muttertiere waren 2013 (1), 2015 (2) und 2016 (3).

Es wurde keine Milch zum menschlichen Verzehr abgegeben. Die Haltung der Tiere erfolgte im Stall auf Tiefstreu mit circa 7 Abteilen, die nach Alter, Geschlecht und Milchleistung getrennt waren. Die Kitze wurden komplett getrennt von den Altziegen gehalten. Zwischen den meisten anderen Boxen konnten sich die Tiere durch die Absperrungen hindurch berühren.

In der Kontrollherde wurden 115 weiße deutsche Edelziegen gehalten. Die Tiere wurden in biologischer Haltung mit Auslauf- und Weidezugang gehalten. Diese Herde wurde in der Vergangenheit regelmäßig serologisch kontrolliert, es gab keine Hinweise auf eine Coxiellose. Schließlich erfolgte 2019 eine Untersuchung aller Ziegen auf Antikörper. Bei keinem Tier konnten Antikörper nachgewiesen werden, d. h. die Wahrscheinlichkeit war hoch, dass es sich um eine vollempfängliche Herde handelte.

### Impfungen

In der Fallherde wurden die Ziegen ab dem 3. Lebensmonat mit einem kommerziell erhältlichen Phase I-Impfstoff geimpft (Coxevac, Ceva Sante Animal, Libourne, France). Die Grundimmunisierung bestand aus 2 Dosen à 2 ml Impfstoff im Abstand von 3 Wochen. Der Impfstoff wurde in einer Hautfalte hinter der Schulter subkutan appliziert. Der Impfstoff verblieb in der Kühlung im Praxisauto und die Impfung erfolgte erst nach der Entnahme aller Proben, um eine Kontamination der Proben durch den Impfstoff zu verhindern.

Im Januar/Februar 2020 erfolgte die Grundimmunisierung aller Jährlinge und adulten Ziegen, die Kitze wurden dann im April 2020 grundimmunisiert. Im Oktober/November wurden alle Ziegen, die sich auf dem Betrieb befanden, einmalig und aus Gründen des Betriebsmanagements in zwei Gruppen revakziniert. Im Mai 2021 wurden die neuen, im Betrieb geborenen Kitze grundimmunisiert, die übrigen Tiere wurden letztmalig einmalig revakziniert. Im Jahr 2022 wurden die neuen Kitze erst im November/Dezember grundimmunisiert. Die Impfung 2023 entfiel aufgrund technischer Probleme, eine Grundimmunisierung der Jahrgänge 2023 und 2024 erfolgte erst im Juni 2024.

Der Tierhalter der Kontrollherde entschied sich aufgrund der Empfänglichkeit seiner Herde für eine prophylaktische Impfung gegen die Coxiellose. Die Grundimmunisierung wurde, wie oben beschrieben, durchgeführt. Die Grundimmunisierung und Impfkontrolle erfolgte in dieser Herde bereits 2019.

### Probenentnahmen

Für die Entnahme von Tupfern wurden Trockentupfer (Abstrichbesteck mit Viskosetupfer, Nerbeplus, Winsen/Luhe, Deutschland) verwendet. Sie wurden nach der trockenen Reinigung der Scham bzw. des Präputiums ca. 3–4 cm tief in die gespreizte Genitalöffnung eingeführt und für ca. 10 Sekunden gedreht. Die Entnahme der Nasentupfer erfolgte entsprechend ohne äußere Reinigung und in beiden Nasenöffnungen.

Mit Umgebungstupfern wurden Staubablagerungen an 8 Entnahmelokalisationen (je 1 Tupfer pro Lokalisation), die sich auf den gesamten Stallbereich verteilten und über den Untersuchungszeitraum konstant gehalten wurden, entnommen. Jeder Tupfer wurde über eine Strecke von ca. 15 cm in der Staubablagerung drehend bewegt, bis er sich grau verfärbte.

Blutproben wurden aus der Vena jugularis (Monovette 9 ml Z, Sarstedt AGCo.KG, Nümbrecht, Deutschland) entnommen. Einzelmilchproben von laktierenden Ziegen wurden nach trockener Reinigung der Zitzen aus beiden Euterhälften in ein 9-ml-Röhrchen mit 5% Borsäure (Kabe Labortechnik GmbH, Nümbrecht, Deutschland) gemolken.

In der Fallherde erfolgte eine Blutprobenentnahme zur Impfkontrolle bei den Altziegen und Kitzen vor und 11 Wochen bzw. 9 Wochen nach der Erstimpfung.

Die Anzahl der entnommenen Proben pro Entnahmezeitpunkt in der Fallherde wurde in Tab. 1 zusammengefasst. In der Kontrollherde wurden Blutproben von 115 Ziegen vor der Grundimmunisierung entnommen. In der 7. Woche nach der Erstimpfung wurden Blut- und Milchproben von 35 Ziegen gewonnen. Es wurden keine Tupferproben entnommen.

**Table:** **Tab. 1** Anzahl von Umgebungs- (UT), Genital- (GT), Nasentupfern (NT), Milch- und Blutproben, die im Verlauf der Überwachung der Fallherde pro Zeitpunkt (Jahr und Monat der Entnahme [JJMM]) entnommen wurden. **Table 1** Numbers of environmental (UT), genital (GT), nasal swabs (NT), milk and blood samples, that were collected per point of time (year and month of sampling [JJMM]) in the case herd.

JJMM	UT	GT	NT	Milch	Blut
2001	8	20	27	14	27
2002	8	21	27	16	n.d.
2004	8	27	27	21	42
2005	8	40	39	20	n.d.
2006	8	39	39	20	39
2007	n.d.	12	12	n.d.	n.d.
2008	n.d.	12	n.d.	4	n.d.
2009	n.d.	35	n.d.	8	n.d.
2010	8	31	n.d.	4	n.d.
2011	8	35	35	20	35
2012	8	35	35	n.d.	35
2105	8	48	47	n.d.	49
2106	n.d.	3	3	23	n.d.
2111	8	n.d.	n.d.	n.d.	n.d.
2112	8	28	28	21	28
2202	n.d.	20	n.d.	n.d.	n.d.
2206	8	n.d.	n.d.	n.d.	n.d.
2211	8	31	31	19	31
2304	8	n.d.	n.d.	n.d.	n.d.
2406	8	38	n.d.	n.d.	38
2508	8	n.d.	n.d.	n.d.	n.d.
∑	128	475	350	190	324

n.d.=nicht durchgeführt; n.d.=not done

### Phasen-spezifischer Antikörper-ELISA

Für den Antikörper-ELISA wurden Blut- und Milchproben zentrifugiert (2000xg, 10 Min, 4°C), Serum bzw. entrahmte Milch wurden im Test eingesetzt.

Die Durchführung des PhI- und PhII-Antikörper ELISA wurde detailliert beschrieben 19 . Testplatten wurden mit PhI- und PhII-Antigen beschichtet, Proben wurden in vier Stufen (Serum 1/100 bis 1/10 000, Milch 1/5 bis 1/5000) in Probenverdünner angesetzt. Gebundene Antikörper wurden mit Protein G-Peroxidase nachgewiesen. Eine stark positive Milchprobe einer Kuh zeigte den maximalen Reaktionswert für jede Phase an, der Endpunkttiter (Kehrwert der Verdünnung) wurde für 20% der positiven Kontrolle automatisch berechnet. Titer von 100 (Serum) und 5 (Milch) wurden negativ bewertet. Alternativ zum Titer wurde das Phasenmuster der Probe bestimmt (PhI ^-^ /PhII ^-^ , PhI ^-^ /PhII ^+^ , PhI ^+^ /PhII ^-^ und PhI ^+^ /PhII ^+^ ).

### Quantitative PCR (qPCR)

Für den Nachweis und die Quantifizierung des *C. burnetii* IS1111 Transposase Gens wurde eine TaqMan real-time PCR verwendet. Bis 2024 wurde das VetMax C. burnetii Absolute Quant Kit (Thermo Fisher Scientific/LSI, Lissieu, Frankreich), ab 2025 das bactotype C. burnetii PCR-Kit (Indical Bioscience GmbH, Leipzig, Deutschland) eingesetzt. Die Vergleichbarkeit der Resultate beider Testkits wurde im Rahmen der Validierung geprüft. Beide Methoden wurden entsprechend der Herstellerangaben durchgeführt. Für die DNA-Extraktion wurde ein kommerzieller Kit (IndiMag Pathogen Kit, Indical Bioscience GmbH, Leipzig, Deutschland) nach Herstellerangaben eingesetzt. Die Erregerkonzentration wurde als C.b./ml bzw. als C.b./Tpf. angegeben, dies erfolgte ungeachtet der Tatsache, dass das IS1111 in einer Kopienzahl von 7–110 vorkommen kann 28 .

### Statistische Analysen

Die Datenauswertung und -darstellung erfolgte mit MedCalc Statistical Software Version 19.6 (MedCalc Software Ltd, Ostend, Belgium; https://www.medcalc.org; 2020). Der Logarithmus der Phasen-spezifischen Antikörpertiter vor und nach der Impfung und die Phasen-spezifischen Titer pro Zeitpunkt wurden mit dem zweiseitigen Wilcoxon-Test für gepaarte Proben verglichen. Für jeden weiteren Vergleich pro Parameter wurde die Irrtumswahrscheinlichkeit p mit 2 multipliziert (Bonferroni-Korrektur). Der Vergleich der PhII-Titer der Alt-, Jungziegen in der Fall- und der Altziegen in der Vergleichsherde nach der Impfung wurde mit dem Kruskal-Wallis-Test durchgeführt. Eine Irrtumswahrscheinlichkeit p<0,05 wurde als signifikant bewertet.

Das Antikörpertiter-Verhältnis von Serum zu Milch wurde für PhI und PhII berechnet, wenn ein Antikörpertiter in beiden Materialien positiv bewertet wurde. Der Vergleich der logarithmierten Verhältnisse der PhI- und PhII-Titer in der Fallherde und der Vergleich der PhII-Verhältnisse in der Fall- und Kontrollherde wurden mit dem zweiseitigen t-Test für unabhängige Proben durchgeführt.

Der Korrelationskoeffizient wurde für logarithmierte Erregernachweise in Nasen- und Genitaltupfern bestimmt. Für den Vergleich der Erregernachweise von Nasen- und Genitaltupfern jeweils mit Umgebungstupfern wurde der geometrische Mittelwert der Erregermenge in Umgebungstupfern pro Termin berechnet. Dieser Wert wurde den Entnahmezeitpunkten von Nasen- bzw. Genitaltupfern zugewiesen. Lag für Entnahmezeitpunkte von Nasen- und Genitaltupfern kein Ergebnis einer zeitgleichen Umfeldtupferentnahme vor, so wurden sie dem Ergebnis der nächsten Umfelduntersuchung zugeordnet.

### Ethik-Aussage

In dieser Publikation wurde ein Fall aus der Routinediagnostik beschrieben. Die Maßnahmen erfolgten, um die Gefährdung von Menschen zu reduzieren. Als Kontrolle wurden Daten aus einem Bestand herangezogen, in dem 2019 im Rahmen der Routine eine Impfung und Impfkontrolle durchgeführt wurde.

## Ergebnisse

### Der Verlauf der Erregernachweise in der Fallherde

**Abb. 1:**
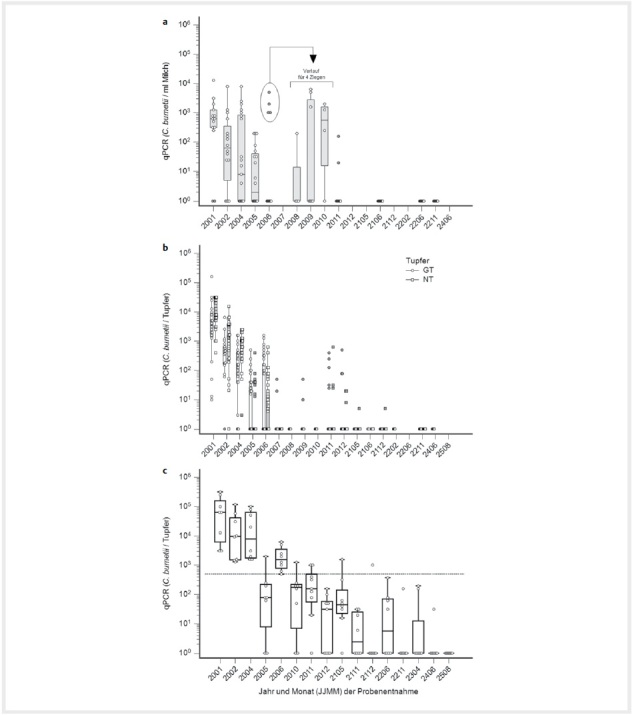
Nachweise von Coxiellen-Genomabschnitten (IS1111) in Einzelmilch ( **a** ), in Genital- (GT) und Nasentupfern (NT) ( **b** ) und in Umgebungstupfern ( **c** ) in einem Coxevac-geimpften Ziegenbestand von Januar 2020 (2001, vor der Impfung) bis August 2025 (2508). Im Zeitraum August–Oktober 2020 wurden die Milchproben- und GT-Entnahmen auf vier Ziegen beschränkt, für die im Juni 2020 der Verdacht auf eine persistente Infektion bestand (wiederholter Coxiellennachweis in der Milch und Phase I-Antikörpertiter>100). Die horizontale Linie ( **c** ) markiert 500 Keime/Tupfer. Quelle: J. Böttcher. **Fig. 1** Detection of genomic sequences (IS1111) of *C. burnetii* in milk ( **a** ), in genital (GT) and nasal swabs (NT) ( **b** ) and in environmental swabs ( **c** ) in a Coxevac-vaccinated goat herd from January 2020 (2001, prior to vaccination) until August 2025 (2508). From August – October 2020 sampling was restricted to four goats for which a persistent infection was suspected (repeated detection of *C. burnetii* and increased PhI-titres). The horizontal line ( **c** ) indicates 500 bacteria/swab. Source: J. Böttcher.

**Abb. 2:**
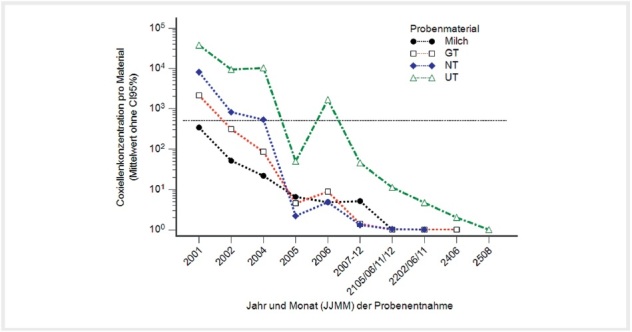
Vergleich der Coxiellennachweise in Milch, Genital-, Nasen- und Umgebungstupfern mit der qPCR im zeitlichen Verlauf. Die horizontale Linie zeigt 500 C.b./Tpf. an. Quelle: J. Böttcher. **Fig. 2** Comparison of the detection of Coxiella by qPCR in milk, genital (GT), nasal (NT) and environmental swabs (UT) over time. The horizontal line indicates 500 C.b./swab. Source: J. Böttcher.

Der Verlauf der Erregernachweise in Milchproben, in Genital- und Nasentupfern und in Umgebungstupfern wurde in den Abb. 1a–c für Einzelproben dargestellt. Die Mittelwerte der Erregerlasten pro Material wurden in Abb. 2 verglichen.

Die Nachweisraten in Umgebungstupfern waren bis April 2020 hoch (24/24), sie verhielten sich über den weiteren Verlauf des Jahres 2020 konstant (31/40), und fielen erst in den Folgejahren sehr langsam ab: 12/24 (2021), 5/16 (2022), 2/8 (2023), 1/8 (2024) und 0/8 (2025). Während der ersten 4 Monate fiel die Erregerlast in den Umgebungsproben nur geringfügig ab. Im Mai wurde vorübergehend eine geringere Konzentration vorgefunden, sie stieg im Juni aber wieder kurzfristig an, um anschließend wieder stetig abzufallen.

Die anfänglich hohen Erregerlasten wurden auch in den einzelnen Probenmaterialien in den ersten Monaten vorgefunden, wobei die mittleren Erregerkonzentrationen in der Reihenfolge Nasen-, Genitaltupfer und Milch abnahmen ( Abb. 2 ). Genabschnitte der Coxiellen konnten in der Milch bis November 2020, in Genitaltupfern bis Dezember 2020 und in 2 Nasentupfern im Mai und Dezember 2021 (je 5 C.b./Tpf.) vorgefunden werden. Der Korrelationskoeffizient (r) für die Erregernachweise in Nasen- und Genitaltupfern betrug 0,766 (CI95%: 0,718–0,807, p<0.0001, 335 Probenpaare). Für Genital- und Nasentupfer im Vergleich zum Mittelwert der Umgebungstupfer betrug er 0,709 (CI95%: 0,661–0,751, p<0,0001, 475 Probenpaare) und 0,8058 (CI95%: 0,766–0,840, p<0,0001, 350 Probenpaare).

Für 4 Ziegen bestand im Juni 2020 der Verdacht auf eine persistente Infektion, da anhaltend hohe Erregerlasten in der Milch vorgefunden worden waren. Im Juni 2021, Juni und November 2022 konnten keine Genabschnitte der Coxiellen in der Milch von laktierenden Ziegen nachgewiesen werden, wobei die verdächtigen Ziegen in dieser Untersuchung enthalten waren.

### Die Impfkontrolle

Die Grundimmunisierung mit Coxevac wurde blutserologisch kontrolliert. Vor der Grundimmunisierung entfielen 11, 6 und 10 adulte Tiere auf die Phasenmuster PhI ^-/^ PhII ^-^ , PhI ^-^ /PhII ^+^ und PhI ^+^ /PhII ^+^ ( Tab. 2 ).

**Table:** **Tab. 2** Veränderung des Musters der Phasen-spezifischen Antikörper gegen *C. burnetii* im Zuge der Grundimmunisierung mit Coxevac. (t0 vor, t1 nach der Grundimmunisierung). **Table 2** Change of the phase-specific antibody pattern against *C. burnetii* in the course of primary vaccination with Coxevac. (t0 prior to, t1 after primary vaccination).

		t0			
		PhI-/PhII-	PhI-/PhII+	PhI+/PhII+	∑
t1	PhI-/PhII+	2	1	0	**3**
	PhI+/PhII-	3	3	2	**8**
	PhI+/PhII+	6	2	8	**16**
	∑	**11**	**6**	**10**	**27**

**Abb. 3:**
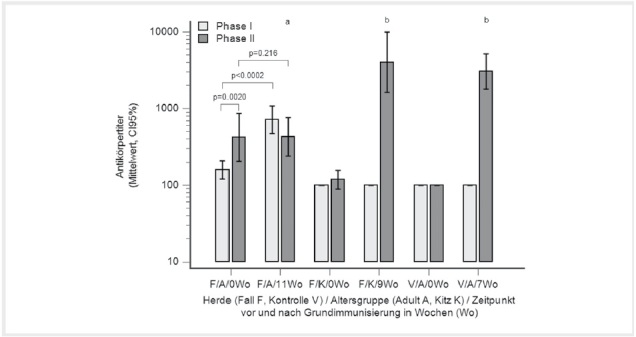
Serologische Kontrolle (Phase I- und Phase II-Antikörper) der Grundimmunisierung mit Coxevac. Adulte Ziegen (n=27) und Kitze (n=13) in der Fall- (F) und Altziegen (n=35) in einer Kontrollherde (V) wurden vor und nach der Grundimmunisierung untersucht. Unterschiedliche Buchstaben zeigen signifikante Unterschiede der Phase II-Titer nach der Grundimmunisierung an (Kruskal-Wallis-Test, p<0,05), ansonsten wurde der Wilcoxon-Test für gepaarte Proben angewandt. Quelle: J. Böttcher. **Fig. 3** Serological verification (phase I- and phase II-antibodies) of the primary vaccination with Coxevac. Adults (n=27) and kids (n=13) in the case (F) and adults (n=35) in a non-infected control herd (V) were tested prior to and after primary vaccination. Different letters are indicating significant differences for PhII-titers following primary vaccination (Kruskal-Wallis-test, p<0.05), otherwise the Wilcoxon-test for paired samples was applied. Source: J. Böttcher.

Die PhII-Titer waren signifikant höher als die PhI-Titer ( Abb. 3 , p=0,002). Nach der Grundimmunisierung wurden 3, 8 und 16 Tiere für die Phasenmuster PhI ^-^ /PhII ^+^ , PhI ^+^ /PhII ^-^ und PhI ^+^ /PhII ^+^ gezählt. Lediglich die PhI-Titer stiegen im Zuge der Impfung signifikant an ( Abb. 3 , p<0,0002). Teilweise fielen die PhII-Titer sogar ab. Je 3 Ziegen wechselten nach der Grundimmunisierung von PhI ^-^ /PhII ^-^ bzw. PhI ^-^ /PhII ^+^ und 2 Ziegen von PhI ^+^ /PhII ^+^ in das Muster PhI ^+^ /PhII ^-^ . Bei 5 von 8 Ziegen mit dem PhI ^+^ /PhII ^-^ -Muster nach der Impfung gingen die PhII-Antikörper in Folge der Impfung verloren. Im Gegensatz zu den Altziegen wurde eine signifikante Zunahme der Phase II-Titer bei grundimmunisierten Kitzen in der Fallherde und auch bei geimpften Altziegen in der seronegativen Kontrollherde beobachtet. Beide Gruppen erreichten ein signifikant höheres Niveau der PhII-Titer im Vergleich zu den Altziegen der Fallherde ( Abb. 3 , p<0,05).

Drei Kitze hatten vor der Impfung schwache PhII-Titer, dennoch stiegen die PhII-Titer nach der Grundimmunisierung an.

**Abb. 4:**
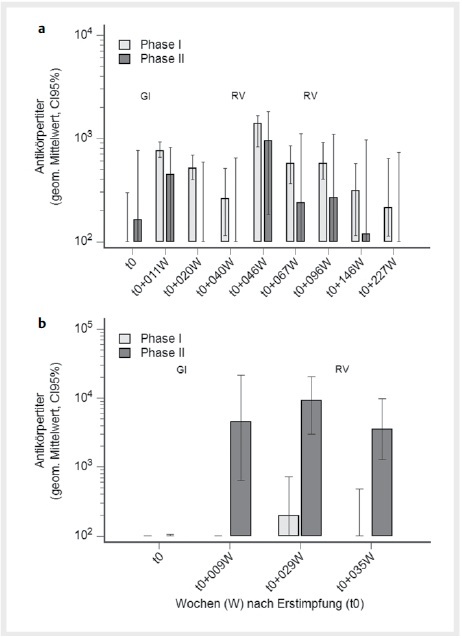
Phase I (PhI) und PhII-Antikörpertiter bei Altziegen ( **a** ) und Kitzen, die in 2020 geboren wurden, nach der Grundimmunisierung (GI) und Revakzination (RV) mit Coxevac. Quelle: J. Böttcher. **Fig. 4** Phase I (PhI) and PhII-antibody titers in adults and kids born in 2020 after primary vaccination (GI) and revaccination (RV) with Coxevac. Source: J. Böttcher.

Die Ziegen wurden im Herbst 2020 revakziniert, für die Altziegen wurde ein signifikanter Anstieg der PhI- (p<0,0001) und PhII-Titer (p=0,0033) im Serum beobachtet. Die Auffrischungsimpfung im Mai 2021 blieb ohne Effekt ( Abb. 4a ). Bei den Jungziegen führte die Revakzination zu keinem Anstieg der bereits hohen PhII-Titer ( Abb. 4b ). Der Anstieg war im Vergleich zum hohen Niveau nach der Grundimmunisierung nicht signifikant. Die Revakzination induzierte ein niedriges Niveau an PhI-Titern (p=0,043).

Das Verhältnis von Serum- und Milchtitern nach der Grundimmunisierung

Die Verhältnisse der Serum- und Milchtiter wurden berechnet. Für den Zeitpunkt vor der Impfung konnten nur 5 und 6, für den Zeitpunkt nach der Grundimmunisierung der Fallherde 20 und 10 für PhI und PhII und für den geimpften negativen Kontrollbestand 34 Verhältnisse für PhII berechnet werden. Aufgrund der geringen Probenzahl wurden die Verhältnisse vor der Grundimmunisierung nicht ausgewertet. Ansonsten wurde in der Fallherde ein mittleres PhI-Verhältnis von 8,3 (CI95% 4,4–13,1) und ein mittleres PhII-Verhältnis von 29,8 (CI95% 12,8–54,6) ermittelt, sie waren signifikant unterschiedlich (p=0,0042). In der Kontrollherde lag das PhII-Verhältnis nach der Grundimmunisierung bei 47,3 (CI95% 40,0–55,9), es war zu den PhII-Verhältnissen in der Fallherde nach Grundimmunisierung signifikant erhöht (p=0,049).

Die Antikörperreaktionen der infizierten Altziegen waren im weiteren Verlauf sehr heterogen, dies deuten die Konfidenzintervalle in der Abb. 4a bereits an. Es traten die unterschiedlichsten Muster auf: Bei einigen Ziegen persistierten hohe Antikörper (PhI und PhII), bei anderen fielen die PhII-Titer schneller ab als die PhI-Titer, so dass sich ein PhI ^+^ /PhII ^-^ -Muster ergab, wiederum andere Tiere verloren die Antikörper über die Zeit komplett.

## Diskussion

Der bislang größte Coxiellose-Ausbruch bei Menschen in den Niederlanden wurde auf eine Zunahme der Milchziegenhaltung und der Bestandsgrößen und auf eine massive Coxiellenausscheidung bei Ziegen zurückgeführt 2 . Obwohl der vorliegende Ziegenbestand relativ klein war, deuteten die Erregerkonzentrationen, die in Vaginaltupfern von 3 Ziegen nachgewiesen wurden, auf ein ernstzunehmendes Infektionsgeschehen hin, insofern war eine intensive Kontrolle des Infektionsverlaufs erforderlich.

### Der Nutzen von Umgebungsproben liegt in der Zukunft

Wir konnten Coxiellen über einen Zeitraum von fast 6 Jahren nach dem Ausbruch mit der qPCR in Umgebungstupfern nachweisen. Der Nutzen derartiger Coxiellennachweise in Umgebungsproben wird durchaus kritisch diskutiert: Tierhalter fürchten die Stigmatisierung ihres Betriebes aufgrund ungerechtfertigter Erregernachweise, die zu einer Meldung führen und Einschränkungen zur Folge haben können. Solange stetig wiederholt wird, dass bereits 1-10 Coxiellen eine Infektion bei Menschen verursachen können 17 , diktiert ein übersteigertes Sicherheitsbedürfnis unser Handeln. Anhand eines Infektionsversuchs konnte bei Meerschweinchen gezeigt werden, dass die Entwicklung von Krankheit dosisabhängig ist 29 . D.h. hohe Erregerlasten sind mit einem höheren Risiko behaftet. Letztendlich werden mit der qPCR DNA-Abschnitte der Coxiellen nachgewiesen, die nicht zwangsläufig eine Infektiosität des Erregers anzeigen. Als die Abortrate in einer Ziegenherde am höchsten war, konnten vermehrungsfähige Coxiellen in Umgebungstupfern nachgewiesen werden, aber bereits 2, 3 und 4 Monate nach der letzten Lammung wurden keine vermehrungsfähigen Erreger mehr in den Umgebungsproben vorgefunden 3 . Damit war die Infektion allerdings nur vorübergehend überstanden, denn in den drei folgenden Lammperioden konnten Coxiellen trotz Impfung wiederum kulturell angezüchtet werden, und mit der qPCR wurden sie sogar über 6 Lammperioden nachgewiesen 14 . D.h. die Infektiosität war mit den Lammperioden assoziiert. Während der ersten 3 Jahre waren lediglich die Jährlinge geimpft, anschließend waren alle Tiere jährlich geimpft worden. Aber trotz der eingeschränkten Impfung in den ersten Jahren sank die Erregerlast bereits im 2. Jahr signifikant ab. Bei geimpften Tieren wurden geringere Erregerlasten im Vergleich zu ungeimpften, und bei Jährlingen geringere Erregerlasten im Vergleich zu multiparen Tieren beobachtet 14 . Dass infektionsempfängliche Jungziegen nach einer Infektion höhere Erregerlasten ausscheiden, und dass ihre Impfung entscheidend ist, wurde bereits gezeigt 30 , 31 . Die verlängerte Persistenz der Infektion trotz Impfung spiegelt sich auch in unserem Beispiel wider: Im 2. Jahr sahen auch wir einen deutlichen Rückgang der Erregerlasten in Umgebungsproben. Die Zeitdauer, in der Genabschnitte der Coxiellen nach einem Ausbruch noch in Umgebungsproben nachweisbar waren, in unserem Fall fast 6 Jahre – schränkt ihre Anwendung zunächst einmal ein. Mit einer quantitativen Bewertung könnte man diesem Problem begegnen: Eine französische Arbeitsgruppe beobachtete ebenfalls schwankende schwach-positive PCR-Ergebnisse 30 . Die Autoren schlugen folgerichtig einen Grenzwert von 500 Coxiellen pro Tupfer (für Einzeltierproben) vor, den wir in der Abb. 1c , 2 aufgenommen haben. Unsere Daten unterstreichen also, dass eine quantitative Bewertung der qPCR-Ergebnisse auch in Hinblick auf eine Risikobewertung sehr wünschenswert wäre.

Eine weitere sinnvolle Anwendung von Umgebungsproben wurde eindrücklich von Kersh et al. 12 beschrieben. Sie analysierten die Verbreitung der Coxiellen innerhalb des Bestandes und aus dem Bestand hinaus. Coxiellen wurden mit dem Schuhwerk sogar bis ins Haus verbreitet. Der Kittelwechsel und Desinfektionswannen für Schuhwerk sind also eine gute Investition.

Wir mussten 5 Jahre warten, bis die Umgebungsproben negativ bewertet wurden. Ab diesem Zeitpunkt können Umgebungstupfer für die Überwachung der Freiheit sehr sinnvoll genutzt werden. Sind Umgebungstupfer positiv, dann ist eine Entnahme von Genital- oder Nasentupfern für eine weitere Abklärung angezeigt. Wir haben die Entwicklung der Erregerausscheidung nach einem Ausbruch beschrieben. Für eine effiziente Nutzung der angeführten Probenmaterialien, sollten wir aber wissen, wie sich die Erregerlasten verhalten, wenn sich ein Ausbruch entwickelt. Hierzu fehlen uns entsprechende Daten, aber Trachsel et al. 13 beschrieben eine Ziegenherde, in der die Umgebungsproben in der qPCR zwar negativ bewertet wurden, während schwach positive Erregerlasten (Ct38,8 und 37,8) in der Milch einer Ziege bzw. in der Plazenta einer weiteren Ziege vorgefunden wurden. Eine ausreichende Kontamination der Umgebung war offensichtlich noch nicht eingetreten.

### Erregernachweise in Nasentupfern

In den Jahren nach dem Ausbruch unterlagen die Erregernachweise in Umgebungstupfern einer gewissen Trägheit. Die Gewissheit eines schnellen Erfolgs der Impfung vermittelten sie nicht. Wir nutzten Nasentupfer unter der Annahme, dass der infektiöse Staub aus der Umgebung kontinuierlich eingeatmet würde, Nasentupfer sollten daher vor dem Hintergrund einer aerogenen Infektion das Infektionsrisiko in einer Herde recht gut widerspiegeln. Bei Schafen sahen wir bereits eine recht gute Übereinstimmung von Nasen- und Vaginaltupfern, die wir in den vorliegenden Untersuchungen bestätigen konnten 15 , 19 . Im Vergleich zu den Umgebungstupfern wurden Coxiellen in Genital- und Nasentupfern weniger lange nachgewiesen, d. h. diese Art der Tupfer ist für die Einschätzung des Bestandes nach einem positiven Ergebnis in Umgebungstupfern sehr sinnvoll. Erregernachweise in Vaginaltupfern setzen wir mit einer Ausscheidung infektiöser Coxiellen gleich, obwohl auch für dieses Probenmaterial ein nicht unerhebliches Kontaminationsrisiko aus der Umgebung besteht 32 , 33 . Demgegenüber gibt es bislang keine Belege, dass eine Erregerausscheidung über die Atemwege erfolgt 1 . Bei Menschen können Coxiellen zwar eine atypische Pneumonie verursachen, aber selbst unter diesen Bedingungen erfolgt keine Infektion von Mensch-zu-Mensch. Ein signifikant (basierend auf den Konfidenzintervallen!) höherer Korrelationskoeffizient zwischen Erregernachweisen in der Nase im Vergleich zu Genitaltupfern und den Mittelwerten der Umgebungstupfer deutet den Einfluss der Umgebung auf die Nachweise auf der Nasenschleimhaut an. Dennoch wurden Coxiellen weniger lange in der Nase im Vergleich zur Umwelt vorgefunden. Wahrscheinlich sind höhere Erregerlasten in der Umgebung für einen Coxiellennachweis auf der Nasenschleimhaut erforderlich. Der Abfall der Erregerlasten in der Umgebung im Mai 2020 (2005) spiegelt sich auch bei den Nasen- und Genitaltupfern wider. Der schnellere Abfall der Erregerlast in Nasentupfern könnte auch damit erklärt werden, dass sich intakte, infektiöse Coxiellen länger als die DNA zerfallener Erreger auf der Nasenschleimhaut halten. Der PCR-Nachweis von Coxiellen auf der Nasenschleimhaut als indirekter Hinweis auf Infektiosität ist eine spannende Hypothese, die aber noch eines Beweises bedarf. Schließlich haben Nasentupfer einen praktischen Vorteil, denn sie sind einfacher zu entnehmen als Vaginal- oder Präputialtupfer.

Der Verlauf der Erregerlasten in den unterschiedlichen Probenmaterialien wurde in der Abb. 2 zusammengefasst. Zu Gunsten der Klarheit wurde auf die Konfidenzintervalle (CI95%) verzichtet. Die Erregerlasten in den Nasentupfern rangierten in den Monaten nach der Infektion auf Platz 2 hinter den Umgebungsproben, sie fielen aber nach einem halben Jahr hinter jene der Genitaltupfer und Milchproben zurück. Auffallend ist der parallele Abfall und der folgende Anstieg in Umgebungs-, Nasen- und Genitaltupfern im Mai und Juni 2020. Dieser deutet daraufhin, dass Genital- und Nasentupfer dem Einfluss der Umgebung unterliegen.

### Die Prädilektionsstelle Euter

Das Euter ist zumindest bei Kühen und in Hinblick auf die Persistenz der Coxiellen eine Prädilektionsstelle. Coxiellen können bei Kühen über längere Zeiträume oder gar lebenslang in der Milch nachgewiesen werden 8 , 9 , 34 , 35 . Die Persistenz des Erregers geht mit erhöhten PhI-Titern einher 8 , 9 . Nach experimenteller Infektion waren Coxiellen in der Milch von Ziegen 38 bis 52 Tage nachweisbar 10 . In einer anderen Untersuchung wurden Coxiellen in der Milch einer Ziege über ein Jahr bei 3 von 4 Untersuchungen nachgewiesen, und es konnte mit Hilfe der in-situ-Hybridisierung bzw. Immunhistochemie gezeigt werden, dass der Erreger sich im Eutergewebe vermehrte 36 . In unserem Fall bestand für vier Ziegen zwar der Verdacht auf eine persistente Infektion. Hierfür sprachen bis in den Mai/Juni 2020 persistierende hohe Erregerlasten in Verbindung mit hohen PhI-Titern (Daten nicht gezeigt). Letztendlich konnte aber bei keinem Tier in der Herde eine über ein Jahr hinausgehende oder gar lebenslange Persistenz im Euter demonstriert werden. Insofern scheint die über ein Jahr hinausgehende persistente Infektion bei Ziegen, eine sehr seltene Ausnahme zu sein.

### Unübliche Antikörpermuster nach der Impfung infizierter Ziegen

Die Infektion führt zunächst zur Bildung von PhII-Antikörpern, PhI-Antikörper wurden verzögert gebildet 25 . Die Dominanz der PhII- gegenüber den PhI-Titern beobachteten auch wir. Aber bei den Altziegen dominierte nach der Grundimmunisierung ein unübliches Antikörpermuster: Sie führte in der Gruppe der Altziegen lediglich zu einem signifikanten Anstieg der PhI-Titer, während die PhII-Titer unbeeinflusst blieben. Teilweise sanken die PhII-Titer im Blutserum ab. Nach der Grundimmunisierung wurde das PhI ^+^ /PhII ^-^ -Antikörpermuster bei 8 von 27 Ziegen gesehen. Im Gegensatz hierzu reagierten die Kitze des Jahrgangs 2020 und die adulten Ziegen im Kontrollbestand, der anhand einer serologischen Stichprobe als wahrscheinlich vollempfänglich eingestuft wurde, erwartungsgemäß, d. h. sie reagierten mit einem sehr starken PhII-Titeranstieg auf die Impfung, während nur vereinzelt schwache PhI-Titer auftraten. Ähnliche Beispiele wurden für Ziegen bereits beschrieben, allerdings wurden andere Phasen-spezifische Tests (ELISA, Immunfluoreszenz-Antikörpertest (IFAT)) eingesetzt 37 , 38 . Auch im Feld wurde bei Ziegen zunächst eine Dominanz der PhII-Antikörper gesehen, die zumindest in einem Fall nach der Impfung in eine Dominanz der PhI-Titer umschlug 27 , 39 . Darüber hinaus wurde eine Dominanz der PhI-Antikörper auch schon vor der Impfung in zwei Ziegenbeständen beobachtet 13 . Das Muster PhI ^+^ /PhII ^-^ wurde selten bei Kühen oder Schafen beobachtet 7 , 15 , insofern war die Häufigkeit dieses Musters bei Ziegen insbesondere nach der Impfung auffällig. Ein derartiges Phasenmuster wurde bislang nicht bei Menschen beobachtet. Uns fehlt eine Erklärung für das Auftreten dieses Musters: Es trat bei Böcken (Daten nicht gezeigt) und bei Altziegen, die vor 2020 geboren wurden, auf. Es lag kein augenscheinlicher Zusammenhang zur Persistenz des Erregers oder der Erregerausscheidung vor. Ein sehr hoher Infektionsdruck könnte zu diesem Phasen-Muster geführt haben. Unter Umständen weist die Präferenz der PhI-Antikörperbildung auf eine eingeschränkte Immunantwort hin. Schließlich ist auch die chronische Infektion des Menschen durch hohe PhI-Titer charakterisiert, aber in diesen Fällen werden gleichzeitig auch immer hohe PhII-Titer gesehen 17 , 40 . Mit den bislang gebräuchlichen kommerziellen Tests, die Antikörper gegen beide Phasen messen 41 , fallen derartige Reaktionsmuster nicht auf. Andeutungsweise ist erkennbar, dass sich die immunologische Reaktion der Ziegen nach einem Ausbruch von jener der Schafe und Rinder unterscheidet. Weitere Abklärungen sollten daher erfolgen.

Die Auffrischungsimpfung führte bei den Altziegen zu einem vorübergehenden Anstieg sowohl der PhI- als auch PhII-Titer, für die 2. Auffrischungsimpfung war dieser Effekt nicht erkennbar. Bei Ziegen scheint eine Auffrischungsimpfung durchaus sinnvoll zu sein 37 , bei Schafen hob die Auffrischungsimpfung zwar die Antikörpertiter und die PhII-spezifische Interferon-γ-Reaktion, aber die Sanierung der Herde gelang allein mit einer Grundimmunisierung der Jungschafe 15 .

### Antikörpernachweise in Serum und Milch

Nicht nur Coxiellen sondern auch Antikörper können in der Milch nachgewiesen werden. Nach der Grundimmunisierung waren die PhII-Titer im Serum um den Faktor 24,8 höher als jene in der Milch, während die PhI-Titer im Serum nur um den Faktor 8,1 höher ausfielen. Dieser Unterschied war signifikant. In der Milch dominiert Immunglobulin G1 (IgG1), die IgG1-Konzentration im Serum von Kühen ist etwa 20fach höher als in der Milch. Dieses Verhältnis ist für IgG2 sogar sehr viel höher (etwa 160) 42 . In unserer Untersuchung fiel das Verhältnis der PhI-Titer niedriger und jenes der PhII-Titer höher im Vergleich zum IgG1-Verhältnis aus. Beim Rind führte die Infektion zu einer deutlichen IgG1-Produktion, während die Impfung mit einem experimentellen Coxiellen-Impfstoff zu einer frühen IgG2-Antikörperbildung gegen Proteinantigene (PhII-Antigene!) führte. Gegen LPS gerichtete IgG2-Antikörper wurden erst sehr viel später (ab der 13. Woche) gebildet 43 , 44 . Da IgG2 nur unzureichend in die Milch sezerniert wird, kann das sehr viel höhere Verhältnis der PhII-Titer möglicherweise mit einer systemischen IgG2-Produktion gegen PhII und einer unzureichenden Sezernierung dieser Antikörper in die Milch erklärt werden. In der Fallherde überlagerten sich die Effekte einer Feldinfektion und der Impfung, während der Effekt in der Kontrollherde allein auf die Impfung zurückzuführen war. In der Tat wurden signifikant höhere Verhältnisse für die PhII-Titer in der Kontrollherde nach der Impfung gemessen. Schließlich könnte die Persistenz der Coxiellen in der Milch auch zu einer lokalen Antikörperproduktion im Euter führen, dieser Effekt würde das Verhältnis der PhI-Titer reduzieren. Im Phasen-spezifischen ELISA wurde ein Protein G-Konjugat eingesetzt, während in den gängigen kommerziellen Tests häufig ein monoklonales Anti-IgG1-Konjugat verwendet wird, um den Nachweis von Serum- und Milchantikörpern zu gewährleisten.

Die IgG1-Produktion wird maßgeblich durch Th2- („humorale Immunität“) und die IgG2-Produktion auch durch Th1-Zellen („zelluläre Immunität“) unterstützt. In dieser Hinsicht gäbe auch das geringere Serum/Milch-Verhältnis der PhII-Titer bei Altziegen in der Fallherde einen indirekten Hinweis auf eine unzureichende zelluläre Immunität, die letztendlich die verlängerte Persistenz und den unzureichenden Effekt der Impfung erklärte.

## Fördermittel

Die Projektfinanzierung erfolgte aus Mitteln des Freistaates Bayern, des Staatsministerium für Ernährung, Landwirtschaft, Forsten und Tourismus sowie der Bayerischen Tierseuchenkasse.

## Danksagung

Die Autoren danken den technischen Mitarbeiterinnen für ihr Engagement und die exzellente Durchführung der Untersuchungen. Den Ziegenhaltern und ihren Familienmitgliedern danken wir recht herzlich für ihr Interesse und ihre Mithilfe.

## Widmung

Diese Arbeit ist Professor Dr. Martin Ganter gewidmet. Wir bedanken uns auf diesem Wege bei einem verdienstvollen Kollegen und Hochschullehrer, für den die Praktikabilität von Maßnahmen stets im Vordergrund stand. Es ist die Praktikabilität, an der sich Gesetze und Verordnungen ihre Ecken und Kanten abstoßen sollten, auf dass sie lebbar werden. Wir wünschen alles Gute für den Ruhestand.

## Notes

FAZIT FÜR DIE PRAXIS Wer die Diagnose der Coxiellose auf die Abortdiagnostik reduziert, vernachlässigt die folgende erhebliche Dauer der Erregerausscheidung. Hohe Erregerlasten erfordern unabhängig vom klinischen Problem Maßnahmen. Die Impfung ist im Ausbruchsfall von geringem Nutzen: Die Immunantwort infizierter Ziegen auf eine Impfung ist eingeschränkt und die Impfung stoppt die Erregerausscheidung nicht kurzfristig. Daher ist die prophylaktische Impfung die einzige Option, die Coxiellose wirkungsvoll zu kontrollieren. In diesem Sinne erfordert die Kontrolle der Coxiellose Weitblick. Freie Herden sind aktuell mit einem Risiko behaftet, denn Neuinfektionen können besonders heftig ausfallen, daher sind freie Herden aktuell nicht erstrebenswert. Umgebungsproben sind nach 5 Jahren der Impfung eine Option, die Freiheit zu überwachen. Positive Ergebnisse in Umgebungstupfern sollten in jedem Fall abgeklärt werden. Geburtsunabhängige Erregernachweise in Nasen- und Vaginaltupfern deuten auf ein aktives Infektionsgeschehen hin. Der Erregerkonzentration in Tupfern sollte in Hinblick auf die Bewertung eine größere Bedeutung beigemessen werden.
